# Supplementary material for: The dorsal aortic compartment is a developmental source of brown adipose tissue in mice
Source: Nat Commun. 2026 Jan 7;17:286. doi: 10.1038/s41467-025-68147-9 (PMC12783139; doi:10.1038/s41467-025-68147-9)
Supplement: Supplementary file 1 — Supplementary Information [file 41467_2025_68147_MOESM1_ESM.pdf]

The dorsal aortic compartment is a source of brown adipose tissue

Sophie Heider, Cornelius Fischer, Ali Kerim Secener, Pedro Vallecillo-Garcia, Georgios Kotsaris, Zarah G. Meisen, Verena Pawolski, Claudia Giesecke-Thiel, Thomas Conrad, Tim J. Schulz, Sascha Sauer and Sigmar Stricker

Supplementary Figures and Tables

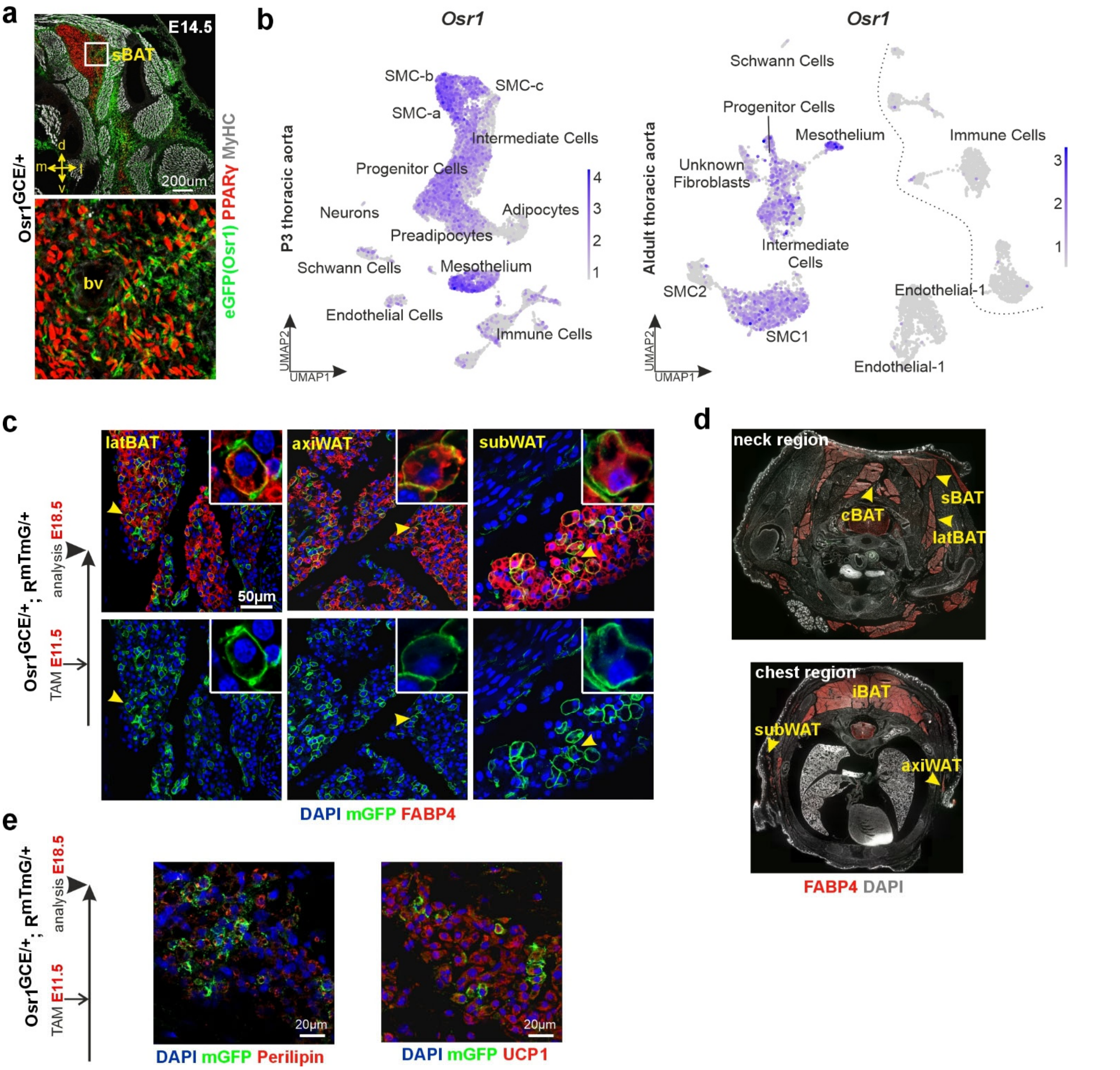

Supplementary Figure 1

a) Immunolabeling of sections of E14.5 Osr1$^{GCE/+}$ embryos for eGFP (Osr1 reporter; green), PPARγ (preadipocytes; red) and MyHC (muscle; white). Boxed regions are shown as magnifications. sBAT: subscapular BAT; bv: blood vessel; representative image of n=4.

b) Feature plots showing *Osr1* expression in single-cell sequencing datasets of P3 and adult thoracic aorta-associated adipose tissue; data cluster annotation taken from Angueira et al. 2021 (Ref. 27).

c) Genetic lineage tracing of E11.5 Osr1+ cells to E18.5 (schematic depiction left) using Osr1$^{GCE/+}$;R$^{mTmG/+}$ embryos; representative images of n=3. Detail images from latBAT: lateral BAT; axilWAT: axillary WAT and subWAT: subcutaneous WAT depots are shown, tissue sections were labeled for mGFP (Osr1 lineage; green), FABP4 (adipocytes; red) and DAPI (nuclei; blue).

d) Overview of adipose tissue depots that develop prenatally; cross sections of E18.5 mouse embryos, adipocytes labelled by FABP4 and DAPI (nuclei; white); representative images of n=3.

e) Genetic lineage tracing of E11.5 Osr1+ cells to E18.5 using Osr1$^{GCE/+}$;R$^{mTmG/+}$ embryos. Detail image from sBAT depot is shown, tissue sections were labeled for mGFP (Osr1 lineage; green), DAPI (nuclei; blue) and Perilipin (red, left image), or UCP1 (red, right image); representative images of n=3 for both panels.

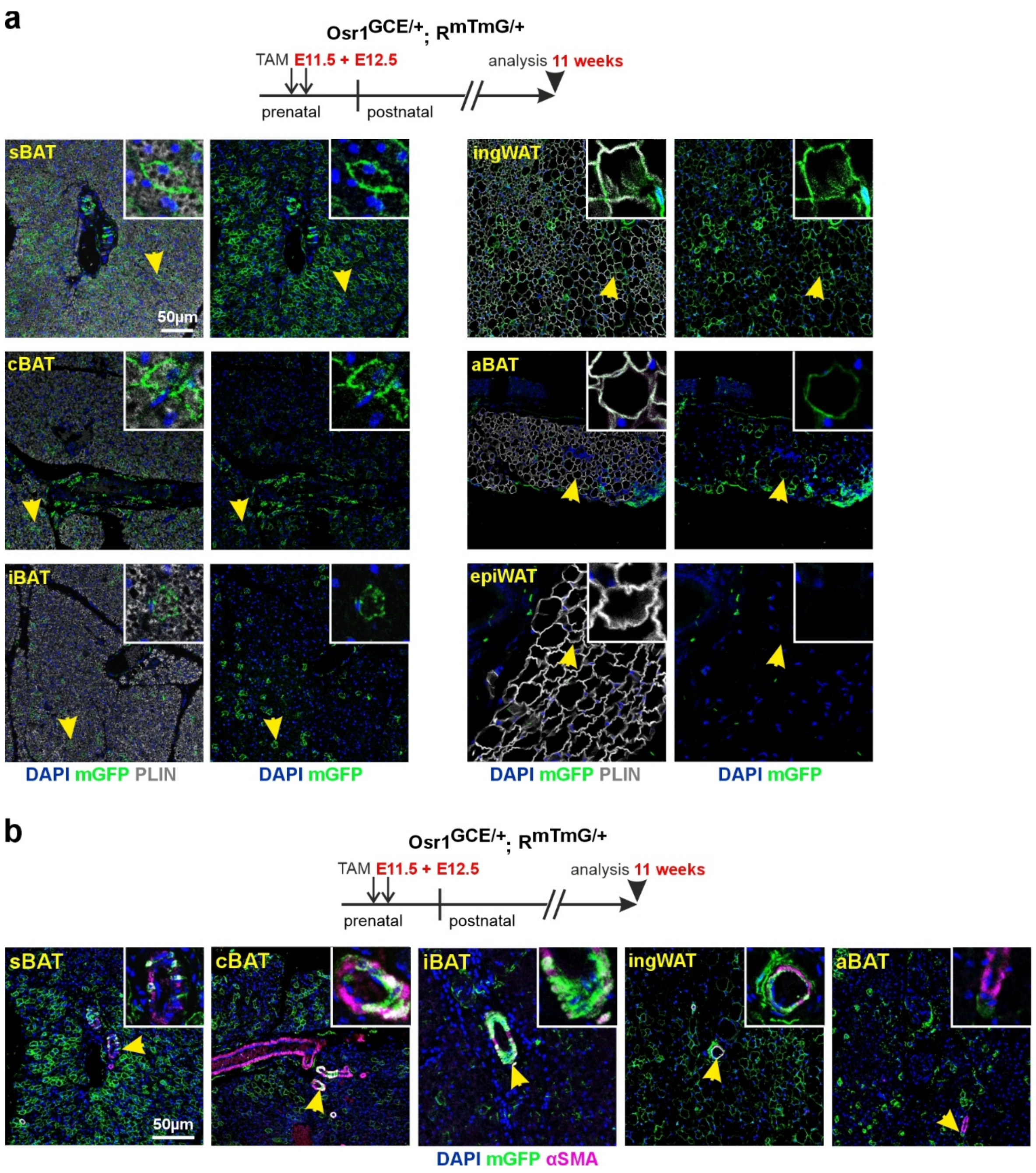

Supplementary Figure 2

a, b) Genetic lineage tracing of E11.5 Osr1+ cells to E18.5 using Osr1$^{GCE/+}$;R$^{mTmG/+}$ embryos. Indicated BAT and WAT depots are shown, tissue sections were labeled for mGFP (Osr1 lineage; green), DAPI (nuclei; blue). and in **a)** Perilipin (adipocytes; white), or in **b)** alpha-smooth muscle actin (aSMA, red). Arrowheads indicate cells shown in magnification inserts. Representative images of n=2 in both panels.

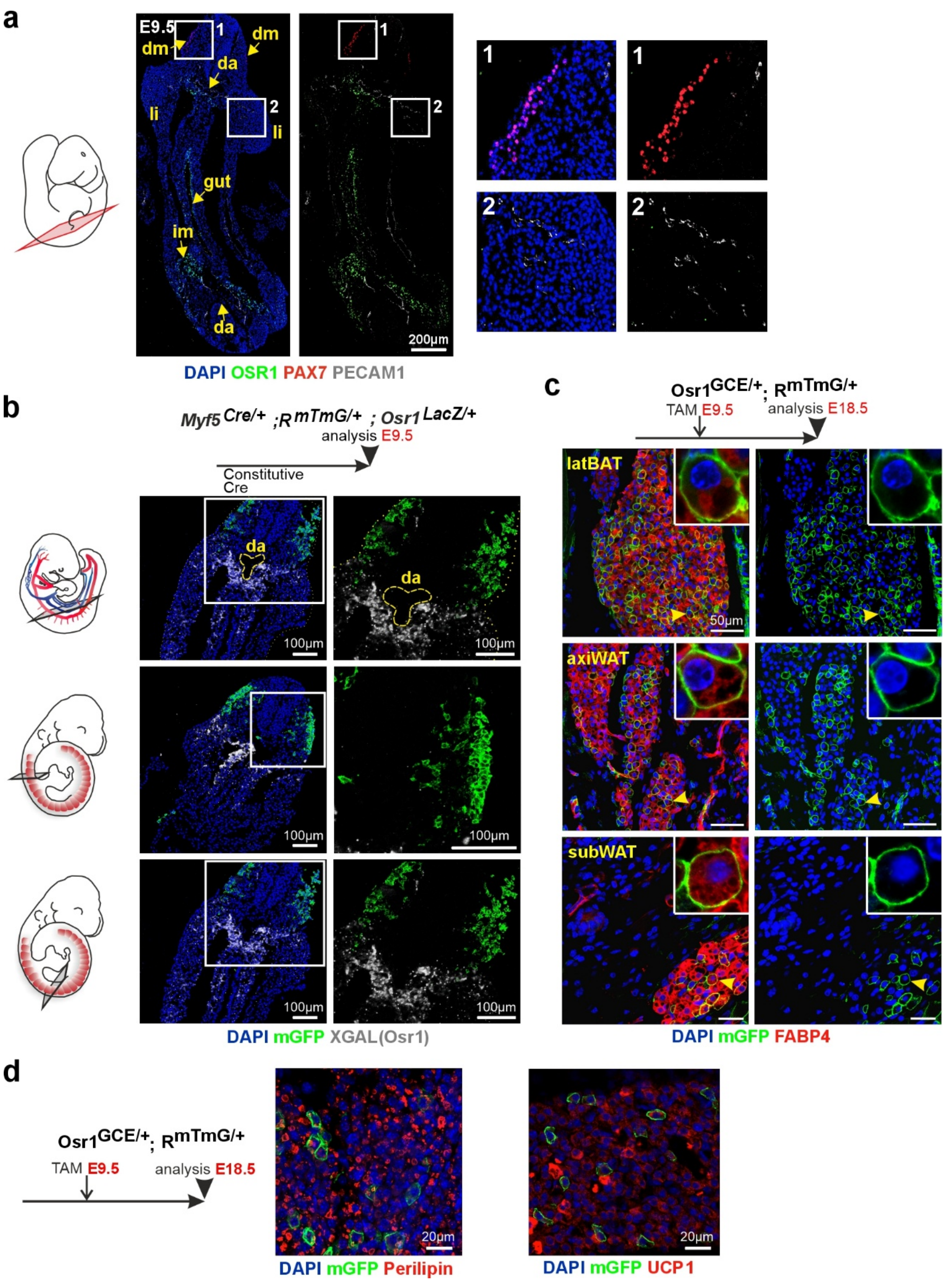

Supplementary Figure 3

a) Section of E9.5 wild type mouse embryos at indicated level labelled for OSR1 (green), PAX7 (red), PECAM1 (white) and DAPI (nuclei; blue); representative image of n=3. Boxed areas (1: somite region; 2: limb bud) shown as magnification right. Abbreviations: da: dorsal aorta; dm: dermomyotome; im: intermediate mesoderm; li: limb buds.

b) Genetic lineage tracing of Myf5+ cells, see schematic depiction. Tissue sections were labeled for mGFP (Myf5 lineage, green), XGAL (Osr1 expression from Osr1-LacZ reporter allele, white) and DAPI (nuclei, blue); representative images of n=3.

c) Genetic lineage tracing of E9.5 Osr1+ cells to E18.5 (schematic depiction left) using $Osr1^{GCE/+}$;$R^{mTmG/+}$ embryos. Indicated BAT and WAT depots are shown, tissue sections were labeled for mGFP (Osr1 lineage; green), FABP4 (adipocytes; red) and DAPI (nuclei; blue). Arrowheads indicate cells shown in magnification inserts; representative images of n=3.

d) Genetic lineage tracing of E9.5 Osr1+ cells to E18.5 using $Osr1^{GCE/+}$;$R^{mTmG/+}$ embryos. Detail image from sBAT depot is shown, tissue sections were labeled for mGFP (Osr1 lineage; green), DAPI (nuclei; blue) and Perilipin (red, left image), or UCP1 (red, right image); representative images of n=3 for both panels.

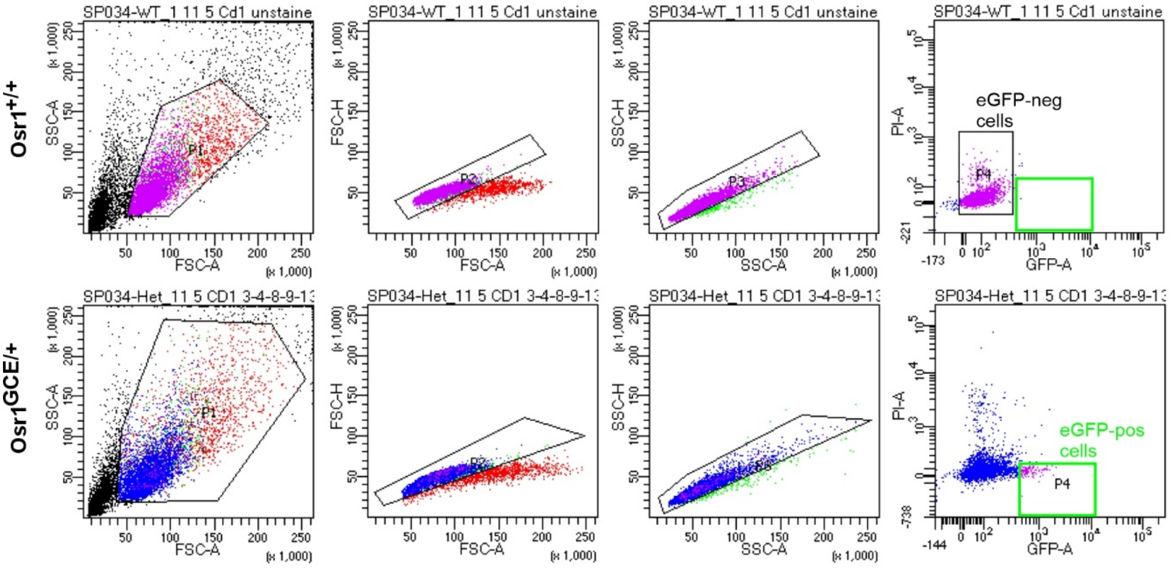

Supplementary Figure 4

a) FACS strategy for isolation of eGFP+ cells from Osr1$^{GCE/+}$ embryos compared to a wild type embryo (Osr1$^{+/+}$) from the same litter.

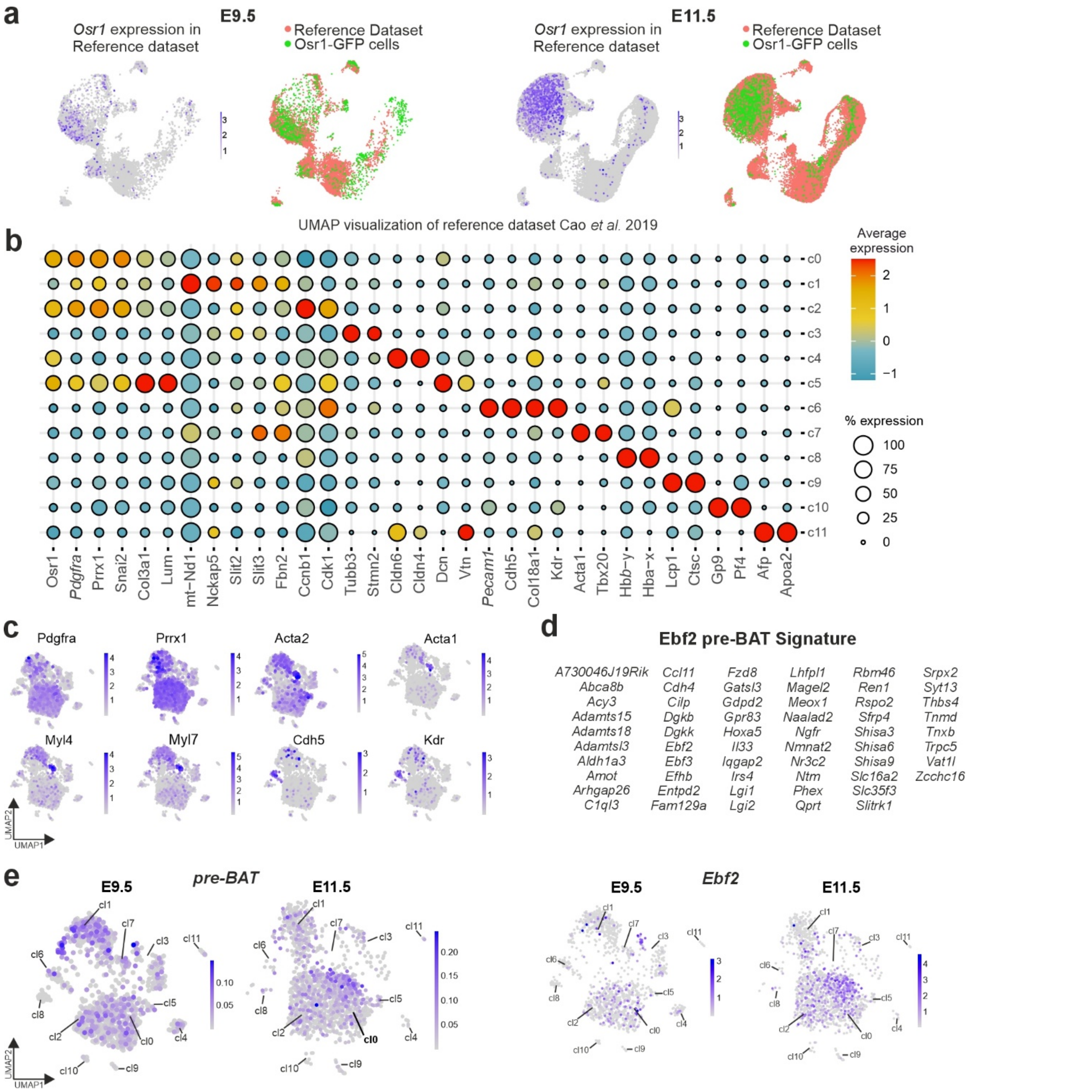

Supplementary Figure 5

a) Feature plots show mapping of Osr1-eGFP single-cell sequencing datasets (E9.5 and E11.5) to the reference whole embryo dataset from Cao et al. 2019, compared to *Osr1* expression in this dataset.

b) Dot plot depiction of cluster-defining marker genes of the combined dataset clusters.

c) Feature plots showing expression of indicated genes in the combined E9.5 and E11.5 Osr1-eGFP dataset.

d) List of genes defining the Ebf2 cell pre-BAT signature (from Wang et al. 2014).

e) Feature plots show mapping of Ebf2 cell pre-BAT signature (left) and *Ebf2* expression (right) in the E9.5 and E11.5 Osr1-eGFP datasets, respectively.

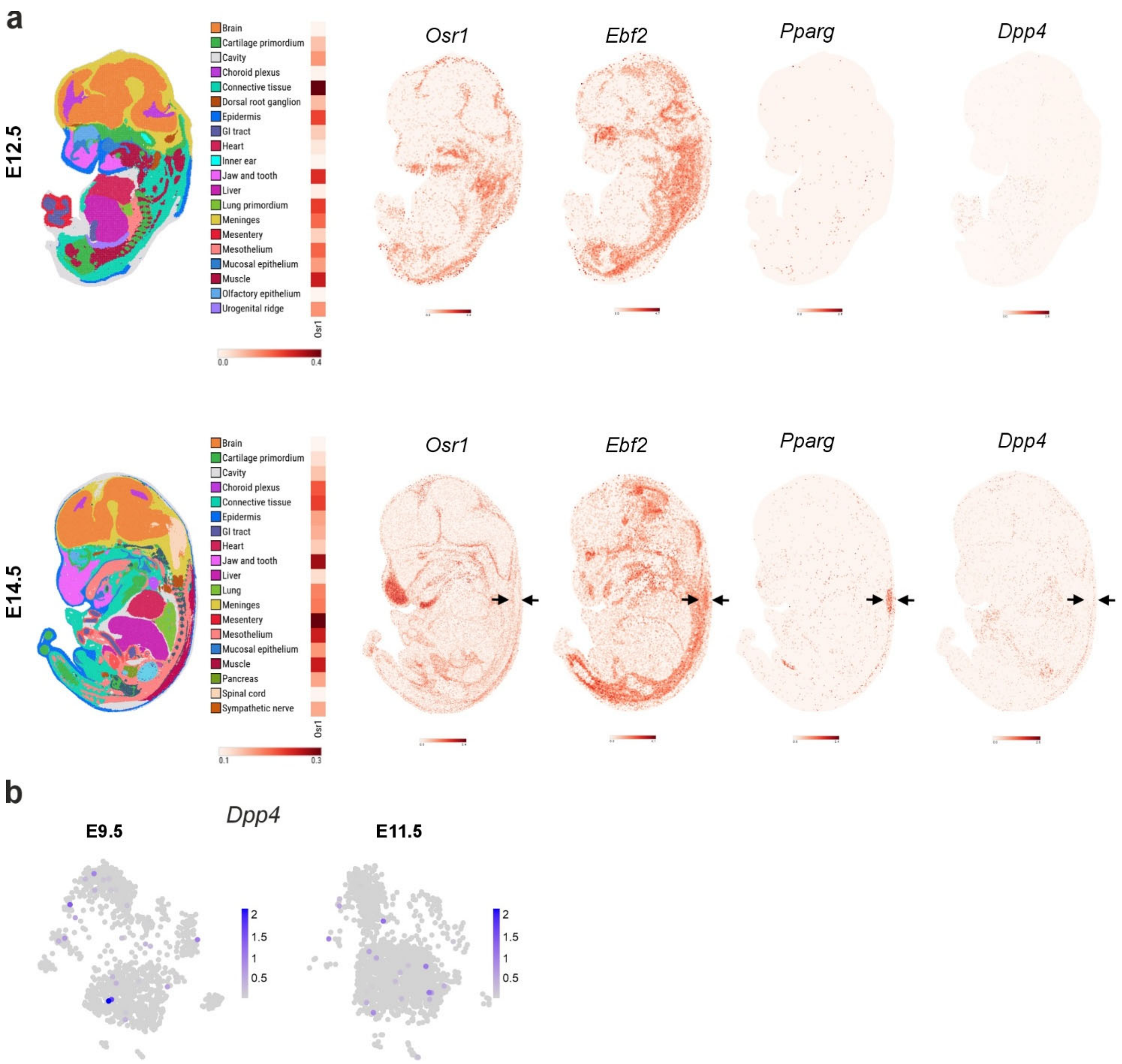

Supplementary Figure 6

a) Mapping of *Osr1* expression on an E12.5 and E14.5 embryo sections of the MOSTA atlas; *Ebf2* and *Pparγ* are shown for comparison. Cell type annotation and heatmap depiction of *Osr1* expression shown left. Arrowheads point out prospective BAT indicated by *Pparγ* expression detectable at E14.5.

b) Feature plots showing expression of *Dpp4* in the E9.5 and E11.5 Osr1-eGFP datasets, respectively.

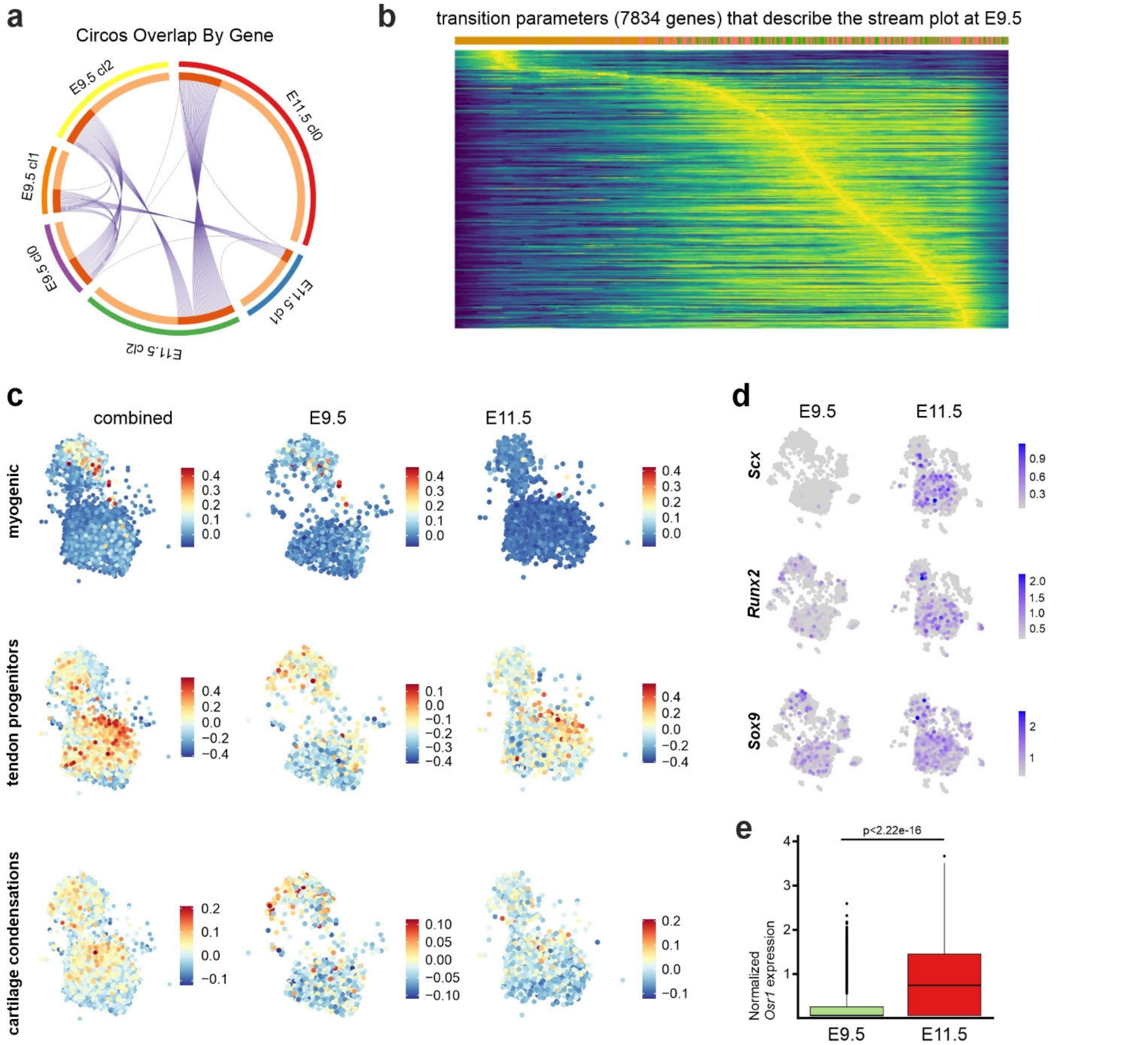

Supplementary Figure 7

a) Metascape based gene overlay analysis compares the cluster enriched genes (2logFC ≥0.5) of cl0, cl1 and cl2 of Osr1-GFP E9.5 and E11.5 datasets.

b) STREAM based generated plot of genes that describe the inferred trajectory of cl1 into cl0 and cl2 within the Osr1-GFP E9.5 dataset

c) Feature plots show module scores of myogenic, tenogenic and chondrogenic progenitor cells derived from Rouco et al. 2021 in the E9.5 and E11.5 Osr1+ cell datasets that were reduced to clusters 0, 1 and 2.

d) Feature plots show gene expression patterns of *Scx*, *Runx2* and *Sox9* in the Osr1+ cell datasets at E9.5 and E11.5

e) Whisker plot depicting normalized *Osr1* expression levels in the E9.5 vs. the E11.5 datasets.

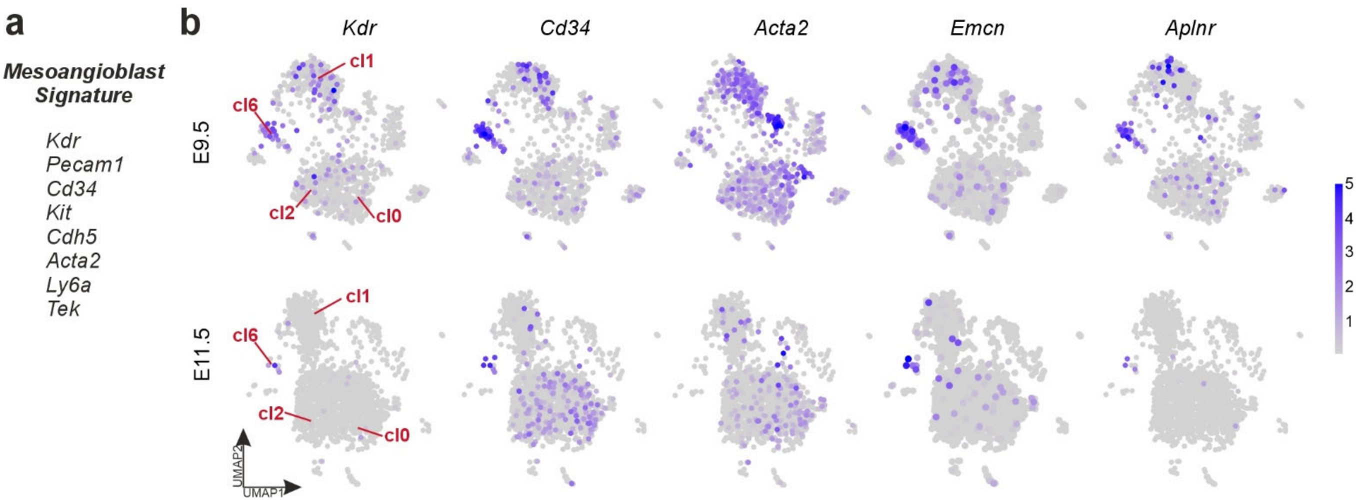

Supplementary Figure 8

a) List of genes describing the mesoangioblast signature.

b) Feature plots showing expression of *Kdr*, *Cd34*, *Acta2*, *Emcn* and *Aplnr* in the E9.5 vs. E11.5 Osr1-eGFP dataset.

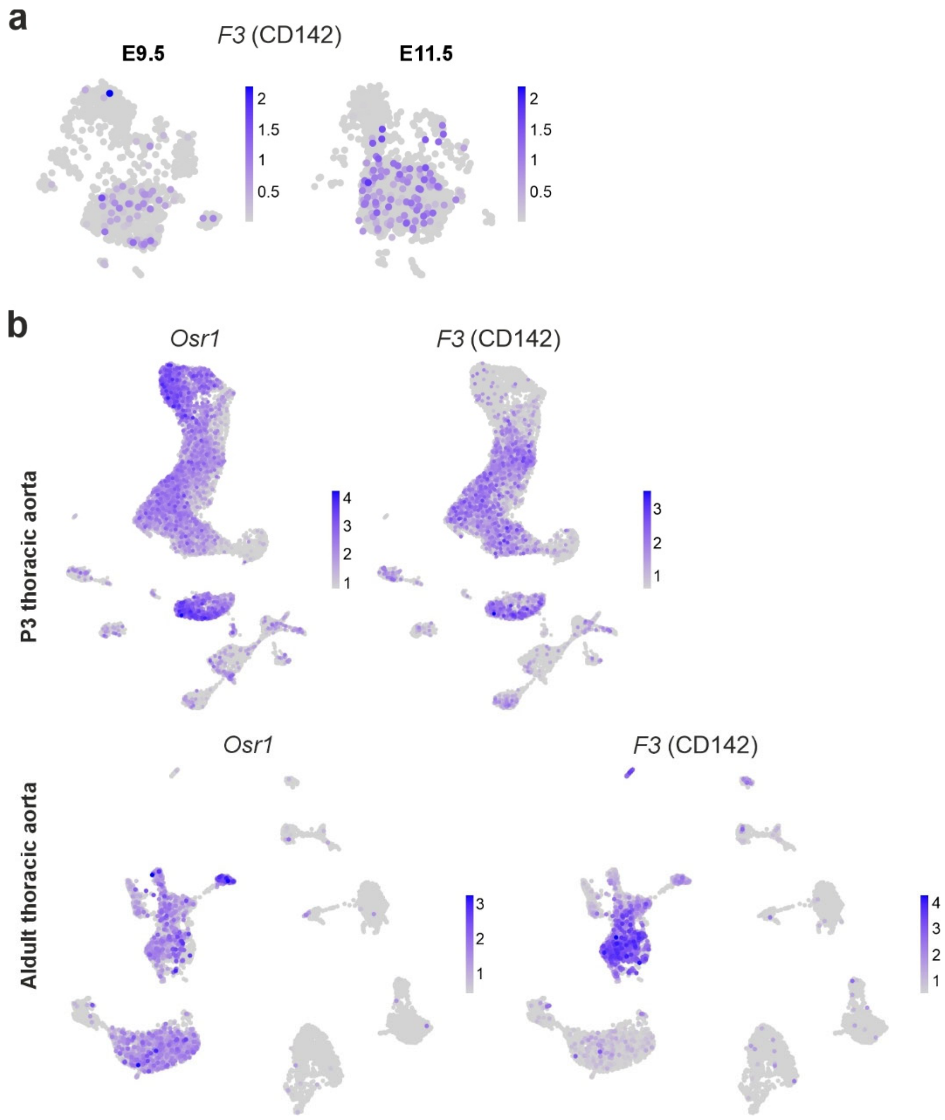

Supplementary Figure 9

a) Feature plots show expression of *F3* (CD142) in the E9.5 and E11.5 Osr1-eGFP datasets, respectively.

b) Feature plots showing *Osr1* and *F3* (CD142) expression in single-cell sequencing datasets of P3 (top) and adult thoracic aorta-associated adipose tissue (bottom); data cluster annotation taken from Angueira et al. 2021 (Ref. 27).

Supplementary Table 1

Antibodies			
name	supplier	catalog	dilution
Rabbit anti-PPARγ	Cell Signaling Technology	Cat# 2443 RRID:AB_823598	1:100
Rabbit anti-OSR1	Sigma-Aldrich	Cat# HPA015525 RRID:AB_1854822	1:100
Chicken anti-GFP	Aves Labs	Cat# GFP-1020 RRID:AB_10000240)	1:800
Rabbit anti-FABP4	Abcam	Cat# ab66682 RRID:AB_1310219	1:300
Guinea pig anti-PAX7	C. Birchmeier, Max Delbrück Centre Berlin	Zhang et al. 2021 doi : 10.1038/s41467-021-21631-4)	1:100
Mouse anti-Myosin Heavy Chain	Millipore	Cat# 05-716-I RRID:AB_309930	1:800
Sheep anti-EBF2	R&D Systems	Cat# AF7006 RRID:AB_10972102	1:20
Hamster anti-PECAM1	DSHB	Cat# 2H8 RRID:AB_2161039	1:250
Rabbit anti-α-SMA	Abcam	Cat# ab5694 RRID:AB_2223021	1:250
Rabbit anti-Perilipin A/B	Sigma-Aldrich	Cat# P1873 RRID:AB_532267	1:300
Rabbit anti-UCP1	Sigma-Aldrich	Cat# U6382 RRID:AB_261838	1:200
Donkey anti-rabbit, Alexa Fluor 488	Thermo Fisher Scientific	Cat# A32790 RRID:AB_2762833	1:500
Donkey anti-rabbit, Alexa Fluor 568	Thermo Fisher Scientific	Cat# A10042 RRID:AB_2534017	1:500
Donkey anti-rabbit, Alexa Fluor 647	Thermo Fisher Scientific	Cat# A32795 RRID:AB_2762835	1:500
Donkey anti-mouse, Alexa Fluor 568	Thermo Fisher Scientific	Cat# A10037 RRID:AB_11180865	1:500
Donkey anti-mouse, Alexa Fluor 680	Thermo Fisher Scientific	Cat# A32788 RRID:AB_2762831	1:500
Goat anti-hamster, Alexa Fluor 568	Thermo Fisher Scientific	Cat# A78960 RRID:AB_2925783	1:500
Goat anti-hamster, Alexa Fluor 647	Thermo Fisher Scientific	Cat# A78962 RRID:AB_2925785	1:500
Goat anti-guinea pig, Alexa Fluor 568,	Thermo Fisher Scientific	Cat# A-11075 RRID:AB_2534119	1:500
Goat anti-guinea pig, Alexa Fluor 647	Thermo Fisher Scientific	Cat# A-21450 RRID:AB_2535867	1:500
Anti-sheep Alexa Fluor 568,	Thermo Fisher Scientific	Cat# A-21099 RRID:AB_2535753	1:500
Goat anti-chicken, Alexa Fluor 488	Thermo Fisher Scientific	Cat# A-11039 RRID:AB_2534096	1:500
