# Supplementary material for: The dorsal aortic compartment is a developmental source of brown adipose tissue in mice
Source: Nat Commun. 2026 Jan 7;17:286. doi: 10.1038/s41467-025-68147-9 (PMC12783139; doi:10.1038/s41467-025-68147-9)
Supplement: Supplementary file 2 — Reporting Summary [file 41467_2025_68147_MOESM2_ESM.pdf]

nature portfolio

Corresponding author(s): Sigmar Stricker

Last updated by author(s): Dec 4, 2025

Reporting Summary

Nature Portfolio wishes to improve the reproducibility of the work that we publish. This form provides structure for consistency and transparency in reporting. For further information on Nature Portfolio policies, see our Editorial Policies and the Editorial Policy Checklist.

Statistics

For all statistical analyses, confirm that the following items are present in the figure legend, table legend, main text, or Methods section.

n/a	Confirmed	
☐	☒	The exact sample size (*n*) for each experimental group/condition, given as a discrete number and unit of measurement
☐	☒	A statement on whether measurements were taken from distinct samples or whether the same sample was measured repeatedly
☐	☒	The statistical test(s) used AND whether they are one- or two-sided<br>*Only common tests should be described solely by name; describe more complex techniques in the Methods section.*
☒	☐	A description of all covariates tested
☒	☐	A description of any assumptions or corrections, such as tests of normality and adjustment for multiple comparisons
☐	☒	A full description of the statistical parameters including central tendency (e.g. means) or other basic estimates (e.g. regression coefficient) AND variation (e.g. standard deviation) or associated estimates of uncertainty (e.g. confidence intervals)
☐	☒	For null hypothesis testing, the test statistic (e.g. *F*, *t*, *r*) with confidence intervals, effect sizes, degrees of freedom and *P* value noted<br>*Give P values as exact values whenever suitable.*
☒	☐	For Bayesian analysis, information on the choice of priors and Markov chain Monte Carlo settings
☒	☐	For hierarchical and complex designs, identification of the appropriate level for tests and full reporting of outcomes
☒	☐	Estimates of effect sizes (e.g. Cohen's *d*, Pearson's *r*), indicating how they were calculated

Our web collection on statistics for biologists contains articles on many of the points above.

Software and code

Policy information about availability of computer code

Data collection	Image acquisition: Zeiss ZEN 3.12, Leica LasX 3.7.0
Data analysis	bcl2fastq v2.20.0; STAR v2.7.3a; Seurat v3; Seurat v4.1.0; velocyto_run, v0.17.17; scVelo v0.2.3; STREAM v1.1; RStudio V 1.2.1335

For manuscripts utilizing custom algorithms or software that are central to the research but not yet described in published literature, software must be made available to editors and reviewers. We strongly encourage code deposition in a community repository (e.g. GitHub). See the Nature Portfolio guidelines for submitting code & software for further information.

Data

Policy information about availability of data

All manuscripts must include a data availability statement. This statement should provide the following information, where applicable:

- Accession codes, unique identifiers, or web links for publicly available datasets
- A description of any restrictions on data availability
- For clinical datasets or third party data, please ensure that the statement adheres to our policy

Single-cell RNA-seq data generated in this study are available via the BioProject accession number PRJNA1186658 [https://www.ncbi.nlm.nih.gov/bioproject/?term=PRJNA1186658]. The processed single cell object is available via Zenodo [https://doi.org/10.5281/zenodo.14170959]. This paper analyzes existing, publicly available data: GSE119945 40 [https://www.ncbi.nlm.nih.gov/geo/query/acc.cgi?acc=GSE119945] and GSE164528 27 [https://www.ncbi.nlm.nih.gov/geo/query/acc.cgi?acc=GSE164528]. All other data generated in this study are provided in the Supplementary Information/Source Data file.

Research involving human participants, their data, or biological material

Policy information about studies with human participants or human data. See also policy information about sex, gender (identity/presentation), and sexual orientation and race, ethnicity and racism.

Reporting on sex and gender	No human participants, data or biological material was used.
Reporting on race, ethnicity, or other socially relevant groupings	No human participants, data or biological material was used.
Population characteristics	No human participants, data or biological material was used.
Recruitment	No human participants, data or biological material was used.
Ethics oversight	No human participants, data or biological material was used.

Note that full information on the approval of the study protocol must also be provided in the manuscript.

Field-specific reporting

Please select the one below that is the best fit for your research. If you are not sure, read the appropriate sections before making your selection.

[x] Life sciences   [ ] Behavioural & social sciences   [ ] Ecological, evolutionary & environmental sciences

For a reference copy of the document with all sections, see nature.com/documents/nr-reporting-summary-flat.pdf

Life sciences study design

All studies must disclose on these points even when the disclosure is negative.

Sample size	Sample sizes are clearly reported in the figure legend. No sample size calculation was performed, as there was no expected effect size. Sample numbers were chosen based on previous experience with Osr1GCE/Rosa26mTmG-based lineage tracing and based on the limited number of mutant mice available. Statistical analysis was performed after data collection.
Data exclusions	No data were excluded
Replication	Quantification is based on 3 independent biological replicates. Representative image data have been observed on at least 2 independent biological replicates. All attempts of replicationhave been successful.
Randomization	Mice were allocated to groups based on genotype. Male and female mice were randomly assigned into the experimental groups.
Blinding	Investigators were not blinded during data collection and analysis as there were no a-priori result expectations. Also, for financial reasons, mouse genotyping, sample preparation and analysis was performed by the same person.

Reporting for specific materials, systems and methods

We require information from authors about some types of materials, experimental systems and methods used in many studies. Here, indicate whether each material, system or method listed is relevant to your study. If you are not sure if a list item applies to your research, read the appropriate section before selecting a response.

Materials & experimental systems

n/a	Involved in the study	
[ ]	[x]	Antibodies
[x]	[ ]	Eukaryotic cell lines
[x]	[ ]	Palaeontology and archaeology
[ ]	[x]	Animals and other organisms
[x]	[ ]	Clinical data
[x]	[ ]	Dual use research of concern
[x]	[ ]	Plants

Methods

n/a	Involved in the study	
[x]	[ ]	ChIP-seq
[x]	[ ]	Flow cytometry
[x]	[ ]	MRI-based neuroimaging

Antibodies

Antibodies used	Commercial antibodies:<br>Rabbit anti-PPARG, Cell Signaling Technology, Cat# 2443, RRID:AB_823598; 1:100<br>Rabbit anti-OSR1, Sigma-Aldrich, Cat# HPA015525, RRID:AB_1854822; 1:100

Chicken anti-GFP, Aves Labs, Cat# GFP-1020, RRID:AB_10000240); 1:800
Rabbit anti-FABP4, Abcam, Cat# ab66682, RRID:AB_1310219; 1:300
Mouse anti-Myosin Heavy Chain, Millipore, Cat# 05-716-I, RRID:AB_309930; 1:800
Sheep anti-EBF2, R&D Systems, Cat# AF7006, RRID:AB_10972102; 1:20
Hamster anti-PECAM1, DSHB, Cat# 2H8, RRID:AB_2161039; 1:250
Rabbit anti-α-SMA, Abcam Cat# ab5694, RRID:AB_2223021; 1:250
Rabbit anti-Perilipin A/B, Sigma-Aldrich, Cat# P1873, RRID:AB_532267; 1:300
Rabbit anti-UCP1, Sigma-Aldrich Cat# U6382; 1:200
Donkey anti-rabbit, Alexa Fluor 488, Thermo Fisher Scientific, Cat# A32790, RRID:AB_2762833; 1:500
Donkey anti-rabbit, Alexa Fluor 568, Thermo Fisher Scientific, Cat# A10042, RRID:AB_2534017; 1:500
Donkey anti-rabbit, Alexa Fluor 647, Thermo Fisher Scientific, Cat# A32795, RRID:AB_2762835; 1:500
Donkey anti-mouse, Alexa Fluor 568, Thermo Fisher Scientific Cat# A10037, RRID:AB_11180865; 1:500
Donkey anti-mouse, Alexa Fluor 680, Thermo Fisher Scientific Cat# A32788, RRID:AB_2762831; 1:500
Goat anti-hamster, Alexa Fluor 568, Thermo Fisher Scientific, Cat# A78960, RRID:AB_2925783; 1:500
Goat anti-hamster, Alexa Fluor 647, Thermo Fisher Scientific, Cat# A78962, RRID:AB_2925785; 1:500
Goat anti-guinea pig, Alexa Fluor 568, Thermo Fisher Scientific, Cat# A-11075, RRID:AB_2534119; 1:500
Goat anti-guinea pig, Alexa Fluor 647, Thermo Fisher Scientific, Cat# A-21450, RRID:AB_2535867; 1:500
Anti-sheep Alexa Fluor 568, Thermo Fisher Scientific, Cat# A-21099, RRID:AB_2535753; 1:500
Goat anti-chicken, Alexa Fluor 488, Thermo Fisher Scientific, Cat# A-11039, RRID:AB_2534096; 1:500
; 1:500
Gifted antibodies:
Guinea pig anti-PAX7, C. Birchmeier, Max Delbrück Centre Berlin; 1:100

Validation

Commercial antibodies: validation available on the company website
Rabbit anti-PPARG, https://www.cellsignal.com/products/primary-antibodies/pparg-81b8-rabbit-mab/2443?srsltid=AfmBOoq9nmhYHlvXoAkkqWtW5dt6Q2hxq5XmqCxHqx2nfAUTr3KmM8v3
Rabbit anti-OSR1, https://www.proteinatlas.org/ENSG00000143867-OSR1/summary/antibody
Chicken anti-GFP, https://www.antibodiesinc.com/products/anti-green-fluorescent-protein-antibody-gfp?utm_source=biocompare&utm_medium=affiliate&utm_campaign=product
Rabbit anti-FABP4, https://www.abcam.com/en-us/products/primary-antibodies/fabp4-antibody-ab66682?srsltid=AfmBOoqeOscD7CKVu6gH9V2-9kmB-ciim8ljMMDbOJ0g8S2fmPeHJP7Z
Mouse anti-Myosin Heavy Chain, https://www.merckmillipore.com/AT/de/product/Anti-Myosin-Heavy-Chain-Antibody-clone-A4.1025,MM_NF-05-716-I-25UL?ReferrerURL=https%3A%2F%2Fwww.google.com%2F
Sheep anti-EBF2, https://www.rndsystems.com/products/human-mouse-ebf-2-antibody_af7006
Hamster anti-PECAM1, https://dshb.biology.uiowa.edu/2H8
Rabbit anti-α-SMA, https://www.abcam.com/en-us/products/primary-antibodies/alpha-smooth-muscle-actin-antibody-ab5694?srsltid=AfmBOoo-ubZJRBMWYOUjObe_NHHnC-6dlxDMqPPuqWjNP1d-cX7vz7u1
Rabbit anti-Perilipin A/B, https://www.sigmaaldrich.com/AT/de/product/sigma/p1873?srsltid=AfmBOorhxx-C3ZpPr1xU0wUuvM7sDKdSLBGI27YzYsejpXvXiAUf_YFt
Rabbit anti-UCP1, https://www.sigmaaldrich.com/AT/de/product/sigma/u6382
Donkey anti-rabbit, Alexa Fluor 488, https://www.thermofisher.com/antibody/product/Donkey-anti-Rabbit-IgG-H-L-Highly-Cross-Adsorbed-Secondary-Antibody-Polyclonal/A32790TR
Donkey anti-rabbit, Alexa Fluor 568, https://www.thermofisher.com/antibody/product/Donkey-anti-Rabbit-IgG-H-L-Highly-Cross-Adsorbed-Secondary-Antibody-Polyclonal/A10042
Donkey anti-rabbit, Alexa Fluor 647, https://www.thermofisher.com/antibody/product/Donkey-anti-Rabbit-IgG-H-L-Highly-Cross-Adsorbed-Secondary-Antibody-Polyclonal/A32795TR
Donkey anti-mouse, Alexa Fluor 568, https://www.thermofisher.com/antibody/product/Donkey-anti-Mouse-IgG-H-L-Highly-Cross-Adsorbed-Secondary-Antibody-Polyclonal/A10037
Donkey anti-mouse, Alexa Fluor 680, https://www.thermofisher.com/antibody/product/Donkey-anti-Mouse-IgG-H-L-Highly-Cross-Adsorbed-Secondary-Antibody-Polyclonal/A32788
Goat anti-hamster, Alexa Fluor 568, https://www.thermofisher.com/antibody/product/Goat-anti-Syrian-Hamster-IgG-H-L-Highly-Cross-Adsorbed-Secondary-Antibody-Polyclonal/A78960
Goat anti-hamster, Alexa Fluor 647, https://www.thermofisher.com/antibody/product/Goat-anti-Syrian-Hamster-IgG-H-L-Highly-Cross-Adsorbed-Secondary-Antibody-Polyclonal/A78962
Goat anti-guinea pig, Alexa Fluor 568, https://www.thermofisher.com/antibody/product/Goat-anti-Guinea-Pig-IgG-H-L-Highly-Cross-Adsorbed-Secondary-Antibody-Polyclonal/A-11075
Goat anti-guinea pig, Alexa Fluor 647, https://www.thermofisher.com/antibody/product/Goat-anti-Guinea-Pig-IgG-H-L-Highly-Cross-Adsorbed-Secondary-Antibody-Polyclonal/A-21450
Anti-sheep Alexa Fluor 568, https://www.thermofisher.com/antibody/product/Goat-anti-Guinea-Pig-IgG-H-L-Highly-Cross-Adsorbed-Secondary-Antibody-Polyclonal/A-21450
Goat anti-chicken, Alexa Fluor 488, https://www.thermofisher.com/antibody/product/Goat-anti-Chicken-IgY-H-L-Secondary-Antibody-Polyclonal/A-11039

Gifted antibodies:
Guinea pig anti-PAX7, Zhang et al. 2021 doi: 10.1038/s41467-021-21631-4

Animals and other research organisms

Policy information about studies involving animals; ARRIVE guidelines recommended for reporting animal research, and Sex and Gender in Research

Laboratory animals	Mouse lines: Osr1tm1(EGFP/cre/ERT2)Amc/J, received from A.P. McMahon, Los Angeles, CA, USA; Gt(ROSA)26Sortm4(ACTB-tdTomato,-EGFP)Luo/J received from A. Kispert, Hannover, Germany. Myf5tm3(cre)Sor received from C. Bichmeier, Gerlin, Germany; B6.Cg-Pax7tm1(cre/ERT2)Gaka/J received from G. Kardon, Salt Lake City, UT, USA. All maintained on a C57Bl6 background. Female mice aged 8-24 weeks were bred for experiments involving embryos; embryonic stages used are indicated in all figure panels. 11-week-old mice were used for all experiments involving adult subjects.
Wild animals	No wild animals were used in this study
Reporting on sex	Male and female mice were assigned into the experimental groups based on genotype, sex-based analysis was not performed due to low animal numbers.
Field-collected samples	No field collected samples were used in this study
Ethics oversight	Animal experiments were performed in accordance with the European Union legislation for the protection of animals used for scientific purposes. Animal maintenance and experimentation was approved by the Landesamt für Gesundheit und Soziales Berlin under license numbers ZH120, G0240/11, G0268/16 and G0250/17. Mouse lines were maintained in an enclosed, pathogen-free facility at a temperature of 22°C, 55% of humidity, and 12 h light / dark cycles.

Note that full information on the approval of the study protocol must also be provided in the manuscript.

Plants

Seed stocks	No plant material was used in thie study
Novel plant genotypes	No plant material was used in thie study
Authentication	No plant material was used in thie study
